# Laugier-Hunziker Syndrome Diagnosed by a Medical Student After Multiple Failed Specialist Evaluations: A Case Report

**DOI:** 10.7759/cureus.104199

**Published:** 2026-02-24

**Authors:** James R Foster, Matthew Murray

**Affiliations:** 1 Family Medicine, University of South Alabama College of Medicine, Mobile, USA

**Keywords:** diagnostic challenge, familial pigmentation, late-onset hyperpigmentation, laugier-hunziker syndrome, oral mucosal pigmentation, primary care diagnosis

## Abstract

Laugier-Hunziker syndrome (LHS) is a rare, benign acquired pigmentary disorder characterized by mucosal macules that can resemble more serious systemic disease. We describe an 82-year-old woman with a four-year history of progressive oral hyperpigmentation involving the lower lip, buccal mucosa, and gingiva, with sparing of the tongue and upper lip, in whom repeated specialist evaluations had not yielded a diagnosis. A comprehensive clinical assessment in a family medicine clinic, including review of prior negative systemic workups, led a third-year medical student to recognize the pattern as LHS. The patient also reported similar pigmentary changes in her mother around the time of a lymphoma diagnosis, raising the possibility of familial or environmental influences, although no causal association could be established. This unusually late-onset presentation underscores the need to consider LHS in elderly patients with unexplained oral pigmentation and highlights the value of attentive history-taking and examination in primary care.

## Introduction

Laugier-Hunziker syndrome (LHS) is a rare, benign, acquired mucocutaneous pigmentation disorder first described by Laugier and Hunziker in 1970 in patients with asymptomatic pigmented macules of the oral mucosa [1]. This syndrome is primarily characterized by hyperpigmentation of the oral mucosa and is frequently associated with longitudinal melanonychia in up to 60% of cases [2-4].

LHS typically affects patients between 40 and 55 years of age and has a female predominance [2-6]. Oral mucosal pigmentation is a common finding among reported cases of LHS [3-5]. The lips are the most frequently affected site, with additional involvement of the buccal mucosa, hard palate, tongue, and gingiva also described [3-5]. Longitudinal melanonychia of the fingernails is commonly reported, and pigmentation may also occur on the hands, feet, face, and genital region [3,4].

This condition is generally sporadic, though rare familial cases have been reported, suggesting a potential inheritance pattern [7]. Despite this, the etiology behind this condition remains unclear, and no identifiable genetic mutations or systemic associations have been identified. 

The clinical significance of LHS lies primarily in its resemblance to Peutz-Jeghers syndrome (PJS), thus requiring the exclusion of associated GI malignancy risks. Other potential and important differentials include Addison’s disease, malignant melanoma, medication-induced pigmentation, smoker’s melanosis, heavy metal exposure, McCune-Albright syndrome, and Carney complex.

We present a rare case of LHS in an 82-year-old woman with a suggestive family history that posed a significant diagnostic challenge despite multiple prior specialist evaluations. This case highlights the importance of comprehensive clinical evaluation, detailed history-taking, and maintaining a broad differential diagnosis when encountering oral mucosal hyperpigmentation.

## Case presentation

Patient information and chief complaint

An 82-year-old Caucasian woman presented to our outpatient family medicine clinic with sinusitis symptoms. During routine examination, extensive oral mucosal pigmentation was noted, prompting further evaluation of this previously undiagnosed condition.

History of present illness

The patient reported a four-year history of progressive, asymptomatic brown-black hyperpigmentation involving the oral mucosa. She stated that no prior clinician had identified the cause of these lesions and that they caused significant distress and embarrassment in public settings. The lesions initially appeared on the lower lip and subsequently extended to involve the buccal mucosa and portions of the gingiva, while sparing the tongue and upper lip. The patient also described painful burning sensations of the mouth and gums that coincided with her gastroesophageal reflux disease (GERD) symptoms, and she noted that the hyperpigmentation appeared more prominent during GERD flares. She denied any ulceration or textural change of the lesions, as well as any melanonychia or additional cutaneous hyperpigmentation.

Past medical history

The patient’s past medical history was significant for chronic sinusitis, GERD without esophagitis, exocrine pancreatic insufficiency, resolved hepatitis B infection with seroconversion, osteoarthritis, depression, and essential hypertension.

Past surgical history

Her surgical history included cholecystectomy, total hysterectomy, bilateral cataract surgery, breast surgery, and a nerve block procedure.

Medications**

Her current medications included fluticasone nasal spray, losartan, pantoprazole, sucralfate, triamcinolone dental paste, “magic mouthwash,” and pancrelipase (Creon).

Family and social history

The family history was notable. Both parents developed malignant lymphoma after prolonged residence near a power plant. Most significantly, the patient reported that her mother developed similar hyperpigmentation localized to the left chest and neck region, which appeared around the time of her lymphoma diagnosis. No other family members were known to have similar pigmentary changes.

Regarding social history, she denied tobacco use, excessive alcohol consumption, and occupational exposure to heavy metals or other potential pigmentation‑inducing substances.

Physical examination

**Figure 1:**
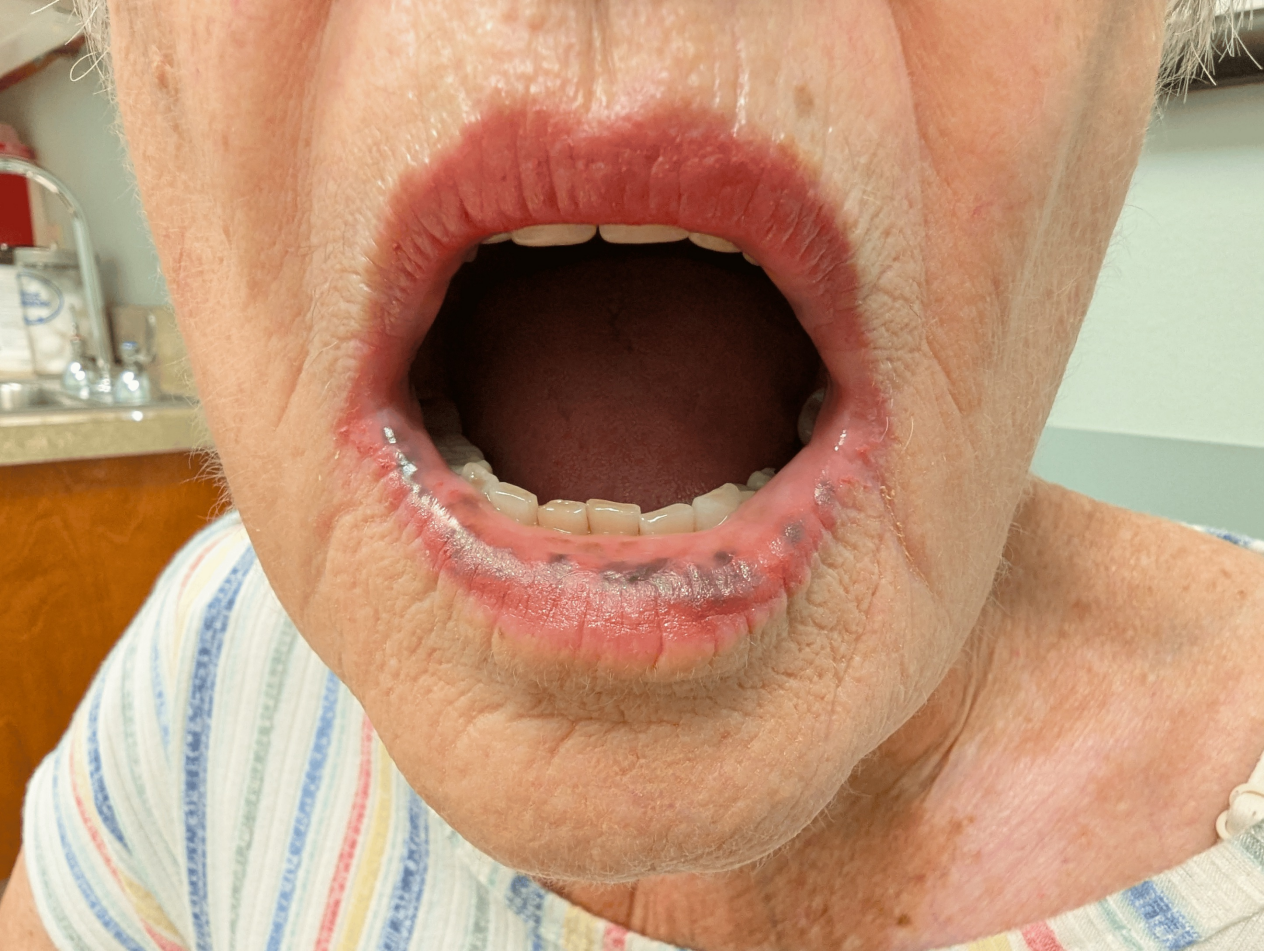
Lower lip mucosal hyperpigmentation in Laugier-Hunziker syndrome This image shows multiple well-demarcated brown to black macules on the lower lip and adjacent oral mucosa.

**Figure 2:**
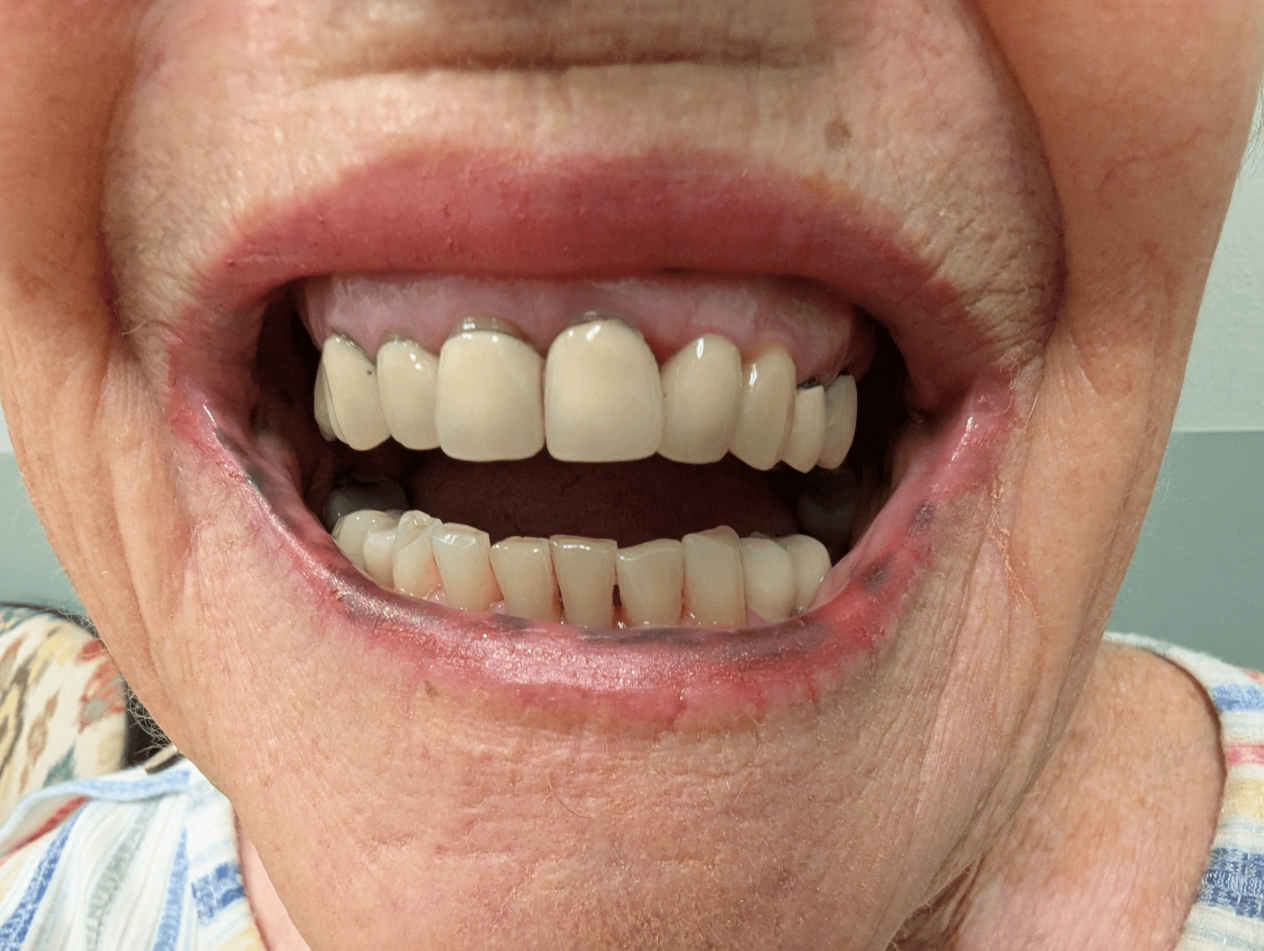
Lower lip macular pigmentation in Laugier-Hunziker syndrome This image shows multiple well-demarcated brown to black pigmented macules on the lower lip.

**Figure 3:**
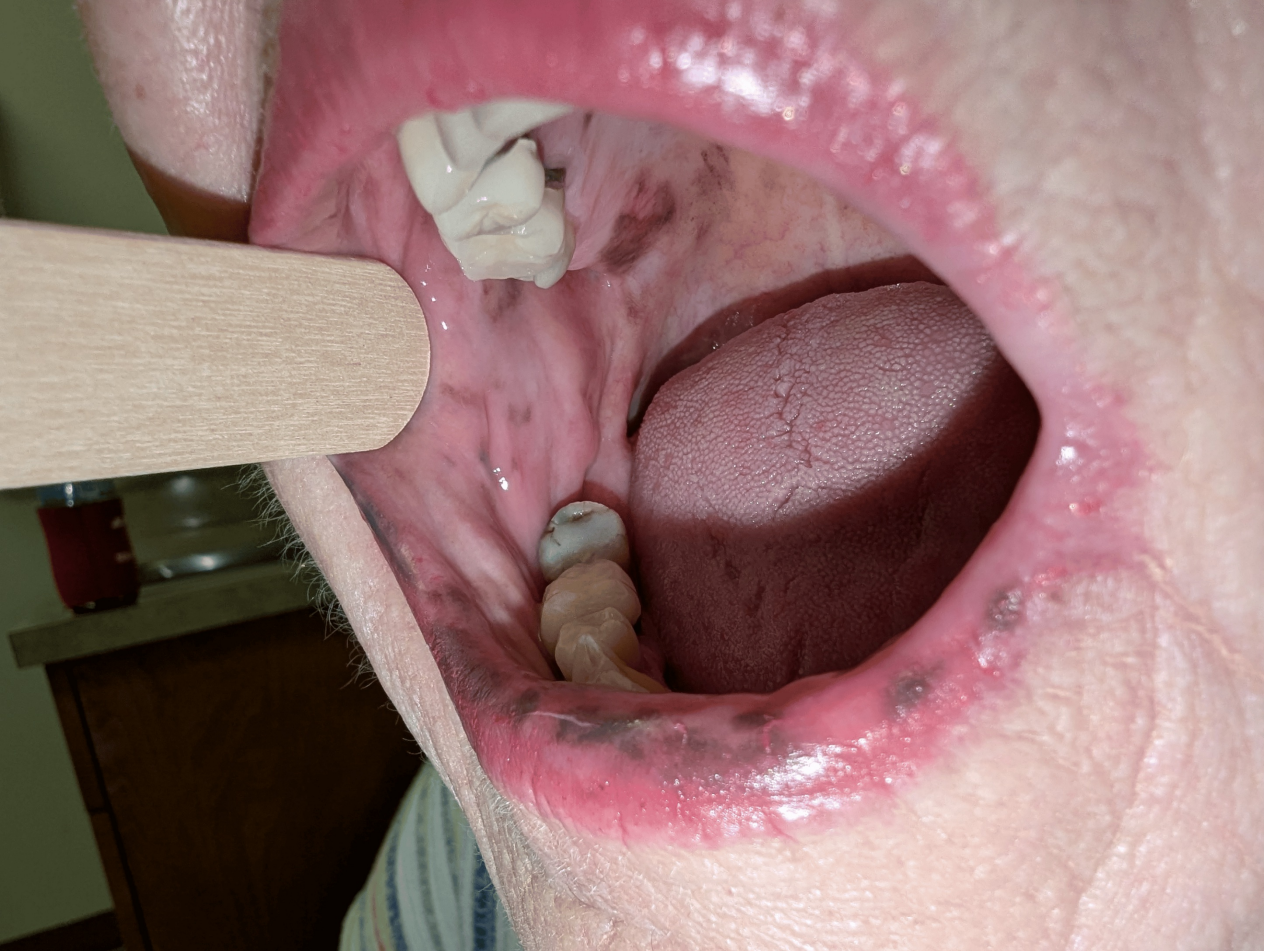
Right buccal mucosal hyperpigmentation in Laugier-Hunziker syndrome This image shows well-demarcated brown to black macular hyperpigmentation of the right buccal mucosa.

**Figure 4:**
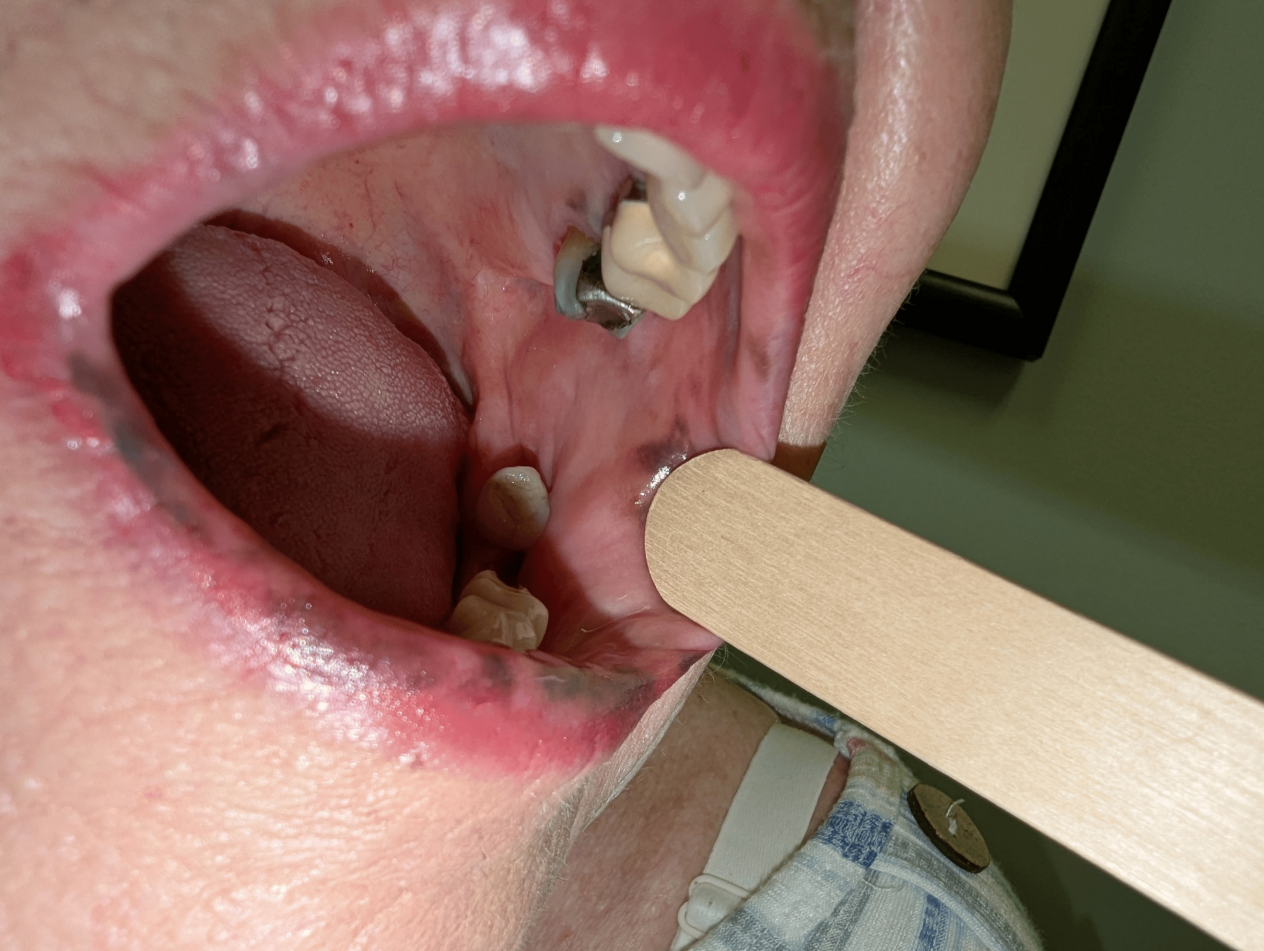
Left buccal mucosal hyperpigmentation in Laugier-Hunziker syndrome This image shows well-demarcated brown to black macular hyperpigmentation of the left buccal mucosa with molar dental amalgams.

**Figure 5:**
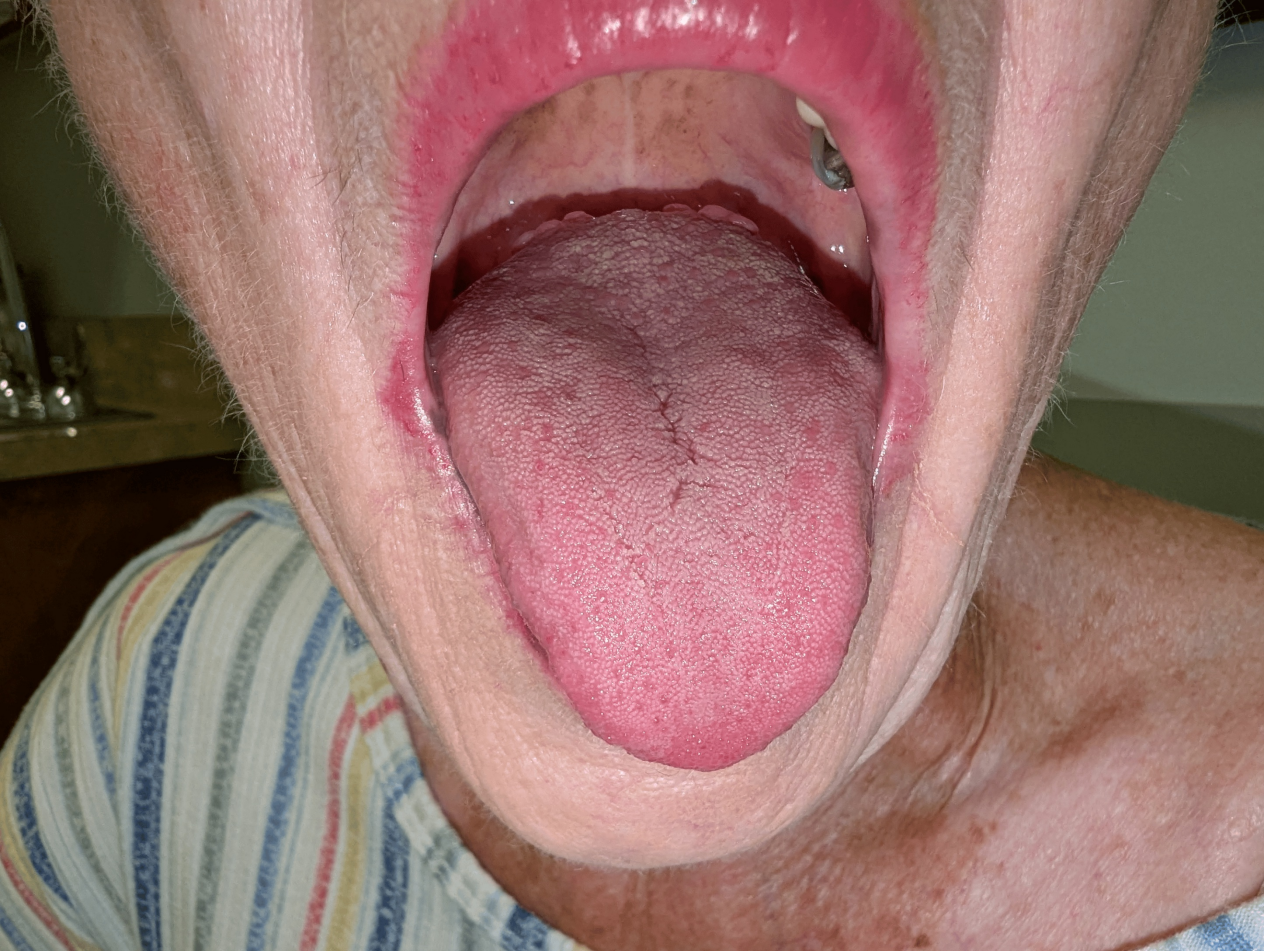
Sparing of the tongue in Laugier-Hunziker syndrome

Figures 1-5 show the physical examination of the patient.

Diagnostic workup

For four years, the patient’s oral hyperpigmentation remained an undiagnosed finding despite consultations with multiple specialists, including dermatology, dentistry, otorhinolaryngology, and gastroenterology. Her hyperpigmentation was overlooked and never officially diagnosed. The diagnosis of LHS was ultimately established in a family medicine clinic by a third-year medical student after prior evaluations had not yielded a unifying explanation. The diagnostic workup in her history is provided below.

Gastrointestinal Evaluation

Two upper endoscopies revealed a short gastric tongue with erythema and non-exudative erosions, a 2 cm hiatal hernia, mild gastritis, and fundic gland polyps. Biopsies were negative for intestinal metaplasia, and she tested negative for *Helicobacter pylori*. Furthermore, the gastric tongue did not meet the criteria for Barrett’s esophagus.

Her previous colonoscopies were unremarkable and only demonstrated benign tubular adenomas, with no evidence of hamartomatous polyps that could potentially indicate PJS.

Imaging Studies

She had various workups related to her cholecystectomy. First, she had a CT of the abdomen/pelvis done for nonspecific gastric pain, which revealed moderate intrahepatic biliary dilatation consistent with normal post-cholecystectomy changes. Additionally, a magnetic resonance cholangiopancreatography (MRCP) confirmed those post-surgical changes with common bile duct dilatation. Neither of these imaging studies found any evidence of masses or other concerning findings.

Laboratory Studies

Hepatitis B serology was significant for natural immunity. Other routine laboratory tests, including comprehensive metabolic panel (CMP), complete blood count (CBC), and prior lipid panels, were well-controlled and within normal limits (WNL).

Histopathological Findings

A biopsy was not performed in our case, given the overall benign clinical appearance and extensive negative systemic workup.

Clinical presentation and differential diagnosis

Our patient’s presentation was classic for LHS. Given the dermatological findings, negative extensive workup, and sporadic onset, this presentation was classic for LHS. Despite this, the distribution pattern and clinical characteristics effectively excluded several important differential diagnoses (Table 1).

**Table 1:** Differential diagnosis and exclusion criteria for oral mucosal hyperpigmentation

Differential diagnosis	Key clinical features	Reason for exclusion in this case
Peutz-Jeghers syndrome	Autosomal dominant; GI polyps; periorificial freckling in childhood	Late onset; no family history; negative GI workup
Addison’s disease	Systemic symptoms (fatigue, hypotension); diffuse pigmentation	No systemic symptoms; well-demarcated macules
Malignant melanoma	Single, irregular, raised/ulcerated lesion; rapid growth	Multiple, stable, flat macules over the years
Dental amalgam tattoo	Solitary, localized blue-grey macule adjacent to amalgam restoration	Inconsistent with dermatological presentation
Medication-induced	Temporal relationship with causative drugs (antimalarials, minocycline).	No such medications in regimen
Smoker’s melanosis	Pigmentation on anterior gingiva; smoker history	Patient is a non-smoker; dermatologic presentation
Heavy metal exposure	Oral discoloration from lead or silver	No exposure by history
McCune-Albright syndrome	Café-au-lait spots, precocious puberty, fibrous dysplasia	Inconsistent with history
Carney complex	Spotty pigmentation, myxomas, endocrine tumors	Inconsistent with history

## Discussion

LHS is a rare, benign, acquired pigmentary disorder that was first described in 1970 in patients with asymptomatic macular hyperpigmentation of the oral mucosa [1]. Since its original description by Laugier and Hunziker [1], fewer than 500 cases have been reported worldwide [4,8]. Reviews of reported cases indicate that LHS occurs predominantly in middle‑aged adults, with mean ages at diagnosis in the late 40s and a female predominance with approximately twice as many women as men affected [2-6]. Clinically, LHS is characterized by multiple brown to black macules on the oral mucosa, most often affecting the lips, buccal mucosa, and hard palate, but also described on the tongue, gingiva, and posterior pharyngeal mucosa [3-5]. Genital macules have been reported on the glans and shaft of the penis in men and on the vulva in women, and some patients also show pigmentary lesions on acral skin, conjunctiva, or perianal mucosa [3-5]. Longitudinal melanonychia is present in up to 60% of reported cases [2-4]. While less likely, cutaneous lesions can also be present on the hands, feet, conjunctiva, oropharynx, and anus [3,4].

Our patient developed mucosal pigmentation at 78 years of age and was only diagnosed at 82, placing her at the extreme older end of the reported age spectrum in the literature and extending the recognized clinical range of LHS into late elderly life. Unlike many reported patients, she had no longitudinal melanonychia or cutaneous involvement, with pigmentation confined to the lower lip and buccal mucosa. 

LHS is regarded as a diagnosis of exclusion [5]. Its recognition requires ruling out systemic disorders that can present with similar mucocutaneous hyperpigmentation, particularly PJS and Addison’s disease [5]. Our patient had various workups across several different specialties, all of which were unremarkable and showed no evidence of hamartomatous polyps, endocrine dysfunction, or malignancies. Despite these various specialist evaluations and extensive negative investigations, her mucosal pigmentation had remained unexplained. The diagnosis was ultimately established in a primary care setting by a third-year medical student.

Being that LHS is a diagnosis of exclusion with a benign course, a biopsy is not routinely necessary. However, when other potential differential diagnoses remain a concern, a biopsy can be warranted and may even be necessary. Biopsies of LHS show a fairly consistent histologic pattern, with increased melanin in basal keratinocytes [9-11]. Pigment incontinence, with melanin present in the superficial lamina propria or papillary dermis within melanophages, is also frequently reported [9,12]. Otherwise, the overlying epithelial architecture is generally unremarkable [9-12].

Furthermore, the number of melanocytes is typically normal or only mildly increased [9,10,12]. This is an important histologic distinguishing feature of LHS compared with the lentiginous melanocytic proliferations, with expansion of the melanocytic population, that characterize the mucocutaneous lentigines of PJS [13]. Because histology in LHS is supportive but not pathognomonic, and our patient’s clinical course and extensive negative systemic workup were characteristic of a benign pattern, we deferred biopsy in favor of clinical diagnosis and longitudinal observation.

LHS is generally considered a sporadic, idiopathic pigmentary disorder without associated systemic findings, malignant predisposition, or familial inheritance patterns. Most published reports note the absence of a clear familial pattern, although a single case report described three affected individuals within one family [7]. Our patient’s history is therefore notable in that both parents developed lymphoma after prolonged residence near a power plant, and her mother reportedly developed similar pigmentary changes on the chest and neck around the time of her lymphoma diagnosis. While there is a difference in anatomical distribution and a lack of systemic disease in our patient compared to the mother, the circumstance remains intriguing. It raises the possibility that host susceptibility and environmental exposures might influence benign pigmentary responses such as those seen in LHS.

Given the benign nature of LHS and the absence of systemic associations or malignant potential, there is no specific medical treatment that is indicated for LHS. For our patient, establishing a definitive benign diagnosis helped reframe her concern from fear of serious disease to a primarily aesthetic issue. In the setting of significant cosmetic distress, there are potential treatment options available. Several reports have described successful lightening of oral and labial macules with Q-switched alexandrite or Nd:YAG laser therapy [12,14,15]. However, these procedures are elective and reserved for selected cases rather than required for disease control.

This case illustrates that LHS can present at an unusually advanced age, may remain unrecognized despite multiple specialist evaluations, and can cause substantial cosmetic distress in the absence of systemic disease. Recognizing the characteristic pattern of oral pigmentation and understanding LHS as a benign diagnosis of exclusion can prevent unnecessary investigations and provide meaningful reassurance to patients. Our experience also highlights the important role that primary care clinicians, including trainees, can play in identifying rare pigmentary disorders when they maintain a broad differential and integrate clinical findings with the existing literature.

## Conclusions

This case expands the known clinical spectrum of LHS, highlighting its potential for onset in the later decades of life. It highlights the need to include LHS in the differential diagnosis of oral mucosal hyperpigmentation, particularly when extensive systemic evaluation is unrevealing. The family history suggests a possible role for genetic predisposition or shared environmental factors in its pathogenesis, although the exact etiology remains unknown. Despite these hypotheses, the true underlying cause, pathophysiology, and complete understanding of LHS are yet to be determined.

Clinically, this case emphasizes the importance of comprehensive history-taking, thorough physical examination, and systematic exclusion of more serious conditions such as PJS and Addison’s disease. It also illustrates how vigilant diagnostic reasoning in a primary care setting, including by trainees, can identify rare conditions that have eluded prior specialist evaluation.
